# In Vitro Study of Composite Cements on Mesenchymal Stem Cells of Palatal Origin

**DOI:** 10.3390/ijms241310911

**Published:** 2023-06-30

**Authors:** Alina Ioana Ardelean, Madalina Florina Dragomir, Marioara Moldovan, Codruta Sarosi, Gertrud Alexandra Paltinean, Emoke Pall, Lucian Barbu Tudoran, Ioan Petean, Liviu Oana

**Affiliations:** 1 Department of Veterinary Surgery, Faculty of Veterinary Medicine, University of Agricultural Sciences and Veterinary Medicine, 3-5 Manastur Street, 400372 Cluj-Napoca, Romania; 2 Raluca Ripan Institute for Research in Chemistry, Babeș-Bolyai University, 30 Fantanele Street, 400294 Cluj-Napoca, Romania; 3 Department of Veterinary Reproduction, Obstetrics and Gynecology, Faculty of Veterinary Medicine, University of Agricultural Sciences and Veterinary Medicine, 3-5 Manastur Street, 400372 Cluj-Napoca, Romania; 4 Faculty of Biology and Geology, Babes-Bolyai University, 44 Gheorghe Bilaşcu Street, 400015 Cluj-Napoca, Romania; 5 National Institute for Research and Development of Isotopic and Molecular Technologies, 65-103 Donath Street, 400293 Cluj-Napoca, Romania; 6 Faculty of Chemistry and Chemical Engineering, Babes-Bolyai University, 11 Arany Janos Street, 400028 Cluj-Napoca, Romania

**Keywords:** dental composite cement, mesenchymal stem cells, biocompatibility

## Abstract

Uniform filler distribution in composites is an important requirement. Therefore, BaO glass, nano hydroxyapatite and quartz filler distribution was realized through PCL microcapsules which progressively release filler during matrix polymerization. Two composites were realized based on a complex matrix containing BisGMA, UDMA, HEMA and PEG400 mixed with a previously described mineral filler: 33% for C1 and 31% for C2. The spreading efficiency was observed via SEM, revealing a complete disintegration of the microcapsules during C1 polymerization, while C2 preserved some microcapsule parts that were well embedded into the matrix beside BaO filler particles; this was confirmed by means of the EDS spectra. Mesenchymal stem cells of palatal origin were cultured on the composites for 1, 3, 5 and 7 days. The alkaline phosphatase (ALP) level was measured at each time interval and the cytotoxicity was tested after 3, 5 and 7 days of co-culture on the composite samples. The SEM investigation showed that both composites allowed for robust proliferation of the cells. The MSC cell pluripotency stage was observed from 1 to 3 days with an average level of ALP of 209.2 u/L for C1 and 193.0 u/L for C2 as well as a spindle cell morphology. Cell differentiation occurred after 5 and 7 days of culture, implied by morphological changes such as flattened, star and rounded shapes, observed via SEM, which were correlated with an increased ALP level (279.4 u/L for C1 and 284.3 u/L for C2). The EDX spectra after 7 days of co-culture revealed increasing amounts of P and Ca close to the hydroxyapatite stoichiometry, indicating the stimulation of the osteoinductive behavior of MSCs by C1 and C2. The MTT assay test showed a cell viability of 98.08% for C1 and 97.33% for C2 after 3 days, proving the increased biocompatibility of the composite samples. The cell viability slightly decreased at 5 and 7 days but the results were still excellent: 89.5% for C1 and 87.3% for C2. Thus, both C1 and C2 are suitable for further in vivo testing.

## 1. Introduction

The restoration or replacement of damaged and diseased parts of body tissues and organs has led to the continuous development of biomaterials in regenerative medicine. This interdisciplinary and revolutionary field has gained major importance due to the contact of new biomaterials with biological systems in recent years. A biomaterial needs to be biocompatible and nontoxic so as to have a positive interaction with the biological system, to be resistant over time and to have mechanical and physicochemical stability [1,2]. A biomaterial must be designed in order to induce specific biological activity [3,4]. The goal of biomaterials consists in generating efficient interactions to treat, repair or replace damaged tissue. The literature characterizes biomaterials as follows: bioinert or biostable materials are named the first generation, biocompatible and bioactive materials are considered the second generation and biodegradable or bioresorbable materials are the third generation while biomimetic or bioinspired materials are the newest fourth generation [5,6].

The biodegradation of a biomaterial is very important, and it should be designed in a way that assures easy absorption by the body without any harmful effects and substitution of the affected tissues while considering sustainability and ecological responsibility [7,8,9]. Some of the newest materials are able to influence the bone healing process and osteogenesis and promote cell differentiation and proliferation of osteoblasts according to previous studies [10,11,12].

The new trend in biomaterial research is their use in dental materials. Their biocompatibility and high chemical stability assure optimal treatment of patients’ pathologies. Scientific research in this field facilitates technological development for biomaterials and assures their successful clinical testing in vivo. Such results help dentists to be trained in new procedures and increase their treatment options to fulfill the particular needs of each patient. Thus, a smart combination of dental biomaterials is required to fulfill these purposes.

Bis-GMA (bisphenol A-glycidyl methacrylate) is a resin used as a base matrix in dental sealant composites due to its good mechanical properties, low volatility, diffusivity in tissues and low polymerization shrinkage [13,14,15,16]. Its adhesion properties strongly depend on its viscosity which is often adjusted using fluidizer additives such as resins containing phosphoric acid residues with methacrylate groups [17]. It is relatively cytotoxic [18] and thus it requires a smart combination with other polymers to enhance the composite’s biocompatibility. Bis-GMA must be substituted with other monomers such as urethane dimethacrylate (UDMA) due to its high viscosity and low degree of conversion. The UDMA substitution increases Bis-GMA’s mechanical properties such as flexural strength, flexural modulus of elasticity and polymerization shrinkage in dental restorative composites [19,20,21].

PEG-400 (polyethylene glycol 400) is a polymer with a low molecular weight that is widely used in a variety of medical formulations such as parenteral, topical, ophthalmic, oral and rectal formulations, mainly due to its low toxicity [22,23]. Glasses such as BaO and BaF_2_ have been utilized due to their capacity to form strong chemical bonds with both soft tissues and hard tissues [15,21], assuring good osteogenic and angiogenic characteristics [24,25] and improving the structural stability and bioactivity of the composite materials [26].

Biomaterial functionalization is a very complex subject requiring the stimulation of an enhanced cell response for osteogenic differentiation [27,28,29]. Size is an important issue for biocompatibility. Since biological structures are nanostructured, the functionalization materials must also be nanostructured for a better physical match. Therefore, particles intended for functionalization must be situated in the size range of 1 to 100 nm [30] and have the ability to increase the material’s biocompatibility [31]. A wide variety of nanoparticles have the potential to inhibit some of the cytotoxic effects of materials destined for biological purposes. Consequently, evaluation of the nanoparticles’ cytotoxic effects during biomaterial development would increase their efficacy and the final products’ safety [32,33].

Dental restorative composites have an important role in dental fissure sealing and enamel surface rehabilitation as well as cavity filling. Their composition might be adapted using colored fillings for mimicking natural teeth color and as a base for glass ionomer cements. Cytotoxic behavior and biocompatibility might be properly adjusted via strict control of their composition, assuring an increase in healing rate [34,35,36].

The current research is focused on the in vitro investigation of the biocompatibility of two dental composite cements. The organic matrix is based on Bis-GMA(2,2-bis[p-(2’-hydroxy-3’-metacryloxypropoxy)phenyl]-propane) doped with UDMA (urethane dimethacrylate) and moderated by HEMA (Hydroxyethyl methacrylate) and PEG 400 (polyethylene glycol). The inorganic matrix consists of mineral filler particles such as BaO glass, nanostructured hydroxyapatite nanoparticles (nHA) and silanized quartz nanoparticles. The novelty element within current research is the controlled release of the mineral filler into the polymer matrix during the polymerization process. Filler-targeted delivery is achieved through polycaprolactone microcapsules containing mineral filler particles assisted by buffalo whey as an anti-agglomerate agent and bioactive promoter. Each of the investigated cements has the same polymeric matrix, and the filler amount differs between C1 and C2 samples.

Mesenchymal stem cells (MSCs) of palatal origin are an optimal testing medium for the biocompatibility of dental materials [37,38,39]. Therefore, both composites were subjected to MSC proliferation aiming at the assessment of biocompatibility and evidence of cytotoxic effects. It is assumed that small differences in the mineral filler ratio might significantly influence the composites’ biologic behavior, in good agreement with the literature [40,41]. The null hypothesis states that a small difference in mineral filler has no influence on the composites’ bioactivity in vitro.

## 2. Results

One of the main goals of the present research is to study the targeted delivery of mineral filler within composite cement samples and its influence on the materials’ biocompatibility regarding MSCs. Therefore, experimental results were grouped according to their specific aspects.

### 2.1. Composite Cement Characterization

**Figure 1:**
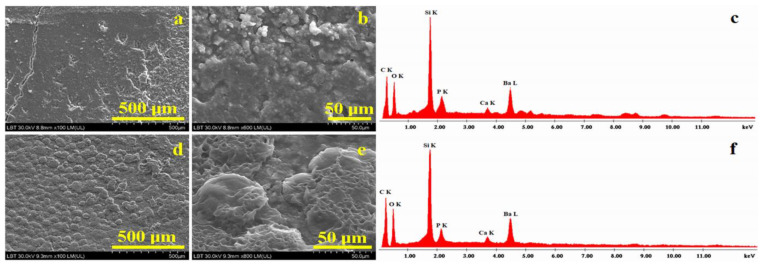
SEM images of composites before cytotoxicity testing: (**a**) C1 overview, (**b**) C1 microstructural details, (**c**) EDX spectrum for details in (**b**), (**d**) C2 overview, (**e**) C2 microstructural details and (**f**) EDX spectrum for details in (**e**).

The microstructural aspect of the composite cement samples is very important and, thus, it was investigated with Scanning Electron Microscopy (SEM). The obtained images and corresponding EDX spectra are presented in Figure 1.

Figure 1a evidences the overview aspect of the C1 composite. The surface topography is not uniform; two morphologies are observed. On the left side, a smooth area with pores and cracks in the composite is observed, and on the right side, an agglomeration of particles is very well defined. The left side crack occurs due to the sample’s superficial excoriation and did not penetrate the composite bulk in depth. High-magnification observation of the C1 composite, Figure 1b, evidences the constituent phases of the sample. In the upper side of the SEM image, the overlapping of particles is evident; barium oxide glass is more evident in this side of the image. They have a boulder shape, and sizes vary from about 1 to 10 μm in diameter. The bottom of the SEM image presents a smoother area where fine particles are more visible and corresponds to nanostructural hydroxyapatite and silanized quartz clusters with rounded shapes and submicron sizes. The elemental composition which resulted for the C1 sample is presented in Table 1. The dominant elements in the surface of the C1 composite are C and O due to the organic matter within the polymer matrix. Si and Ba belong to the nanostructural silica and barium glass filler particles, respectively. Small amounts of P and Ca also occur due to nanostructured hydroxyapatite; their amounts are close to the stoichiometry proportion of nHA.

The microstructural aspects of the C2 composite, Figure 1d, shows the overall morphology of the filler component distribution in the mesoporous structure; it is most likely caused by polycaprolactone microcapsule remains, which are further covered by the polymeric matrix after the composite’s polymerization. The mesoporous microcapsules have an average diameter between 40–120 µm (as observed in Figure 1c) and are evenly placed on the surface of the sample. Microstructural details observed in Figure 1e show with accuracy the filler microcapsule’s disintegration, releasing filler particles that are embedded into the polymeric matrix. The microcapsules have an average diameter around of 55–60 µm. BaO glass particles have a boulder shape and diameter situated around 2–10 μm, while nHA and quartz nanoparticles clusters are less visible except for the smoother area in the upper central side of the SEM image in Figure 1e. Their aspect is rounded, and sizes are situated in the submicron domain. The elemental composition was investigated via EDX spectrometry, and the obtained values are presented in Table 1. The dominant elements are carbon and oxygen due to the polymer matrix. The filler particles’ characteristic elements such as Si, Ba, P and Ca are evidenced, but their amount is less than the one observed in C1, a fact which is in good agreement with the material preparation receipt. 

**Figure 2:**
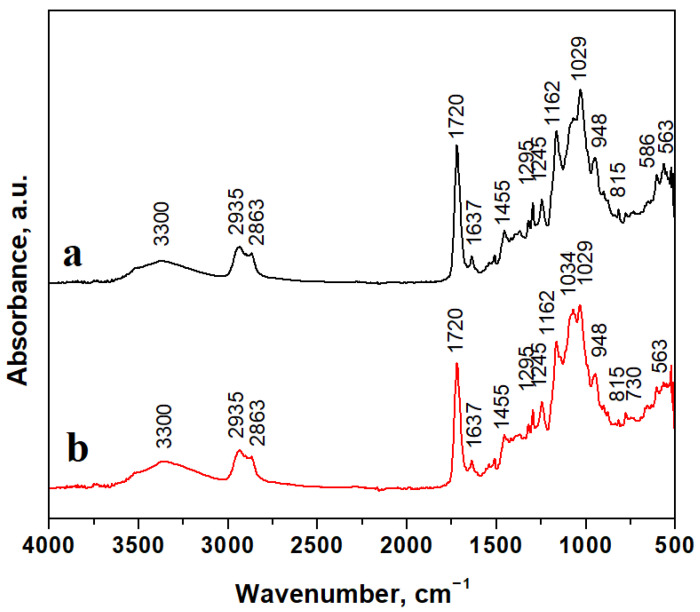
FTIR spectra for composite samples: (**a**) C1 and (**b**) C2.

The chemical bonds and molecular interaction within the polymer and those within the mineral filler were investigated via Fourier Transform Infrared Spectroscopy (FTIR), and the obtained results are presented in Figure 2.

Mineral filler particles are embedded well into the polymer matrix, and FTIR spectra evidence their chemical bonds’ specific vibrations. The absorption band at 586 cm^−1^ belongs to the Ba–O stretching vibration and the band at 730 cm^−1^ also belongs to the Ba-O bond [42,43]. BaO-related absorption bands are stronger for the C1 sample due to a higher filler amount than in the C2 sample. Hydroxyapatite presence is evidenced by intense absorption bands around 560–600 cm^−1^ and 1000–1100 cm^−1^ belonging to the PO_4_ chemical bond, and the absorption band at 3300 cm^−1^ corresponds to adsorbed water. Hence, hydroxyapatite is a calcium carbonate mineral. The absorption band related to the CO_3_ chemical bond appears at 1455 cm^−1^ in good agreement with data in the literature [44].

Silanized quartz particles are evidenced by silicate-specific absorption bands at 1034 cm^−1^, belonging to in-plane Si-O stretching; 815 cm^−1^ belongs to the Si-O stretching of quartz [45,46,47]. The polymer matrix is very well evidenced by specific chemical bonds such as 1162 cm^−1^, related to C-O stretching; 1455 cm^−1^, related to C-H bending; 1367 cm^−1^, related to C=C stretching; 1720 cm^−1^, related to C=O stretching within carboxylic units of Bis-GMA. Broad and less intense bands at 2863 cm^−1^ belong to C-H stretching, and those at and 2935 cm^−1^ belong to O-H stretching within carboxylic groups. The evidenced absorption bands correspond to our previous observation regarding similar compositions [48,49].

### 2.2. MSC Proliferation Morphology

**Figure 3:**
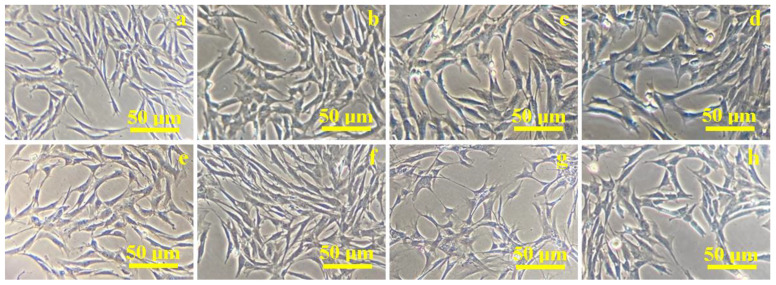
Optical microscopy images of mesenchymal stem cells proliferation on the composite sample C1: (**a**) 1 day, (**b**) 3 days, (**c**) 5 days, (**d**) 7 days; sample C2: (**e**) 1 day, (**f**) 3 days, (**g**) 5 days, (**h**) 7 days.

Cell proliferation evolution over time on the composite samples exposed to a culture medium was first investigated via optical microscopy. The obtained images are presented in Figure 3.

Cells derived from the palatal tissue have spindle-shaped, fibroblast-like morphology with pronounced bipolarity as observed after the first day of proliferation, shown in Figure 3a,e. Changes in their shape are observed after 3 days from the addition of the composites to the cell co-culture, as shown in Figure 3b,f. Some of the cells in the monolayer are flattened and a small proportion of the cells became round, but a high percentage of the cells in the culture retained their spindle-shaped morphology. This stage indicates cellular differentiation. Thus, the cells showed a marked heterogeneity after 5 days of culture: round cells and a high percentage of starred-shape cells are identified beside spindle-shaped ones (Figure 3c,g). They are predominantly organized in local clusters of 10–15 cells. The number of cell clusters is higher for the C2 composite (Figure 3g) than for C1 composite (Figure 3c). Cell differentiation is more evident; after 7 days of co-culture, they are more flattened, and the starred and round shapes are more evident than spindle shapes, as observed in Figure 3d for the C1 sample and in Figure 3h for C2. Both composites prove their ability to induce cell differentiation starting with 5 days and continuing to 7 days of co-culture. It further requires a more enhanced microscopic technique to investigate cell body morphologic aspects regarding the composite surface. 

**Figure 4:**
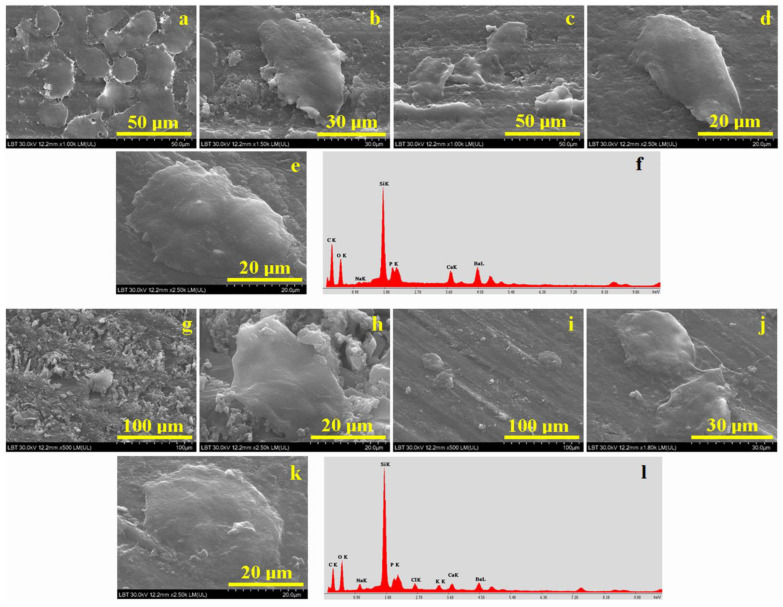
SEM images of composites after cytotoxicity testing for C1: (**a**) 3-day overview, (**b**) 3-day cell details, (**c**) 5-day overview, (**d**) 5-day cell details, (**e**) 7-day cell details and (**f**) EDX spectrum for C1 with cells at 7 days; C2: (**g**) 3-day overview, (**h**) 3-day cell details, (**i**) 5-day overview, (**j**) 5-day cell details, (**k**) 7-day cell details and (**l**) EDX spectrum for C2 with cells at 7 days.

The aspect of C1 and C2 composites’ microstructure visualized via SEM microscopy after MTT assay cytotoxicity testing is given in Figure 4.

Some representative microphotographs of the obtained results are given after 3, 5 and 7 days of MSC cell proliferation. SEM investigation was effectuated in the shortest possible time from the composite samples’ removal from the co-culture medium, and they were not gold sputtered and thus examined in low-vacuum mode. Even with these precautions, only the cell body is predominantly observed, and their terminal pseudopods are almost invisible in Figure 4.

The SEM image from Figure 4a shows the change that occurs in the structure of the C1 composite after 3 days of exposure in co-culture. It can be observed that a group of cells with different sizes is placed randomly and with a flattened structure and rounded or starred shapes. It most likely represents the central body of the MSCs, and the prolonged terminals are not visible due to their thin consistency. The average width of the cells is situated between 15–30 µm and their average length between 20–40 µm. At higher magnification (Figure 3b) a single elongated cell was focalized in the image observation field; its width is about 33 µm and its length is about 52 µm. 

After 5 days of exposure, as shown in Figure 4c, only a few cells are observed. However, it was possible to highlight a single cell at higher magnification (Figure 4d) that has an elongated shape, a width of 20 µm and a length of 36 µm, a fact sustaining cell differentiation. We remark an increase of the viable cell body and a significant reduction of the cell number in the image’s observation field. Cell morphology is significantly changed after 7 days of co-culture on the C1 surface. Figure 4e shows how its length is about 58 µm and its width is 35 µm, a fact which is in good agreement with the optical microscopy in Figure 3d. It sustains a strong differentiation of the cells on the C1 surface after 7 days of proliferation along with potential osteogenic enhancement. This fact is sustained by a small increase in the Ca and P amounts detected in the EDX spectrum in Figure 4f compared to the initial composition of the C1 sample in Figure 1c. The identified amounts of P and Ca correspond to the hydroxyapatite stoichiometry. Significant amounts of Si and Ba were detected as a consequence of filler presence. The complete elemental composition evidenced by the EDX spectrum in Figure 4f is presented in Table 2.

A rough structure of the C2 composite surface is evident after 3 days of exposure, as shown in Figure 4g. It deals with a complex morphological convolution between rough aspects of the C2 composite surface, generated by the polycaprolactone fragments that are still visible on the surface, and cell proliferation, as cells prefer to adhere on average rough surfaces. Thus, several cell clusters are visible in Figure 4g, especially the one most evident in the center of the image. This aspect of the sample surface may be due to cell clusters that overlapped with each other. However, at high magnification, as shown in Figure 4h, a single cell was evidenced. The central body of the cell is clearly visible, having a width of 27 µm and a length of 45 µm. The prolonged terminations of the cell are not visible due to their thin consistency and to the particular investigation conditions within the SEM device.

The morphological aspect of the C2 composite after 5 days of exposure in the culture medium (Figure 4i) shows about three cells surrounded by a smooth surface of the sample compared to the one observed after 3 days of exposure. Thus, two well-defined cells attached to the surface of the composite can be observed at high magnification in Figure 4j, proving the cell differentiation is in good agreement with the optical microscopy observation. Their body is well defined and presents a length of about 30 μm and a width of about 25 μm.

Cell differentiation is more evident after 7 days of co-culture, as shown in Figure 4k. It shows a small increase of the cell body, having a slightly elongated shape with dimensions of 48 μm length and 30 μm width. The corresponding EDX composition is presented in Table 1. Identified carbon and oxygen amounts belong to the organic matter within the composite sample and the cell’s living body. Filler presence is evidenced by the significant amounts of Si and Ba. There is a significant increase in the P and Ca amounts noticed, respecting the stoichiometry proportion within hydroxyapatite. It indicates a strong cell differentiation after 7 days of co-culture induced by the composite sample that facilitates the osteogenic behavior of the cells. 

### 2.3. Biocompatibility Assessment

Biocompatibility is a relatively abstract term describing the ability of a material to be accepted by living tissue without generating adverse reactions. It must be assessed according to specific indicators that allow a proper quantification of the biological effect. 

**Figure 5:**
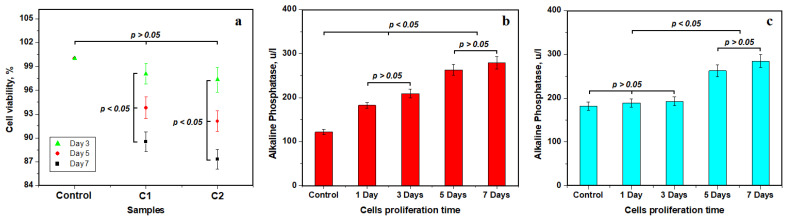
Biocompatibility test results—mean values of three distinct determinations: (**a**) MTT assay cell viability, (**b**) alkaline phosphatase level for C1 sample and (**c**) alkaline phosphatase level for C2 sample.

Therefore, the cytotoxic effect of the investigated composites was assessed via MTT assay using mesenchymal stem cells of palatal origin. This colorimetric assay is based on the reduction of tetrazolium salts to purple formazan crystals by cells that are metabolically active. Viable cells contain NAD(P)H-dependent oxidoreductase enzymes that reduce MTT to formazan. Insoluble formazan crystals were dissolved with dimethylsulfoxide (DMSO), and the absorbance of the chromogenic reaction was evaluated via spectrophotometry at a wavelength of 450 nm. The control sample evidences total cell viability, as shown in Figure 5a. Composite sample C1 evidences an average cell viability of 98.08%, and the C2 sample evidences a mean cell viability of 97.33%. Both values indicate good biocompatibility of the experimental composite cements after 3 days of co-culture, and the statistical analysis shows no significant differences. Cell viability was also tested after 5 and 7 days of co-culture, showing a slow decreased tendency that indicates significant statistical differences. However, the values of 89.5 for C1 and 87.3 for C2 are excellent from the cytotoxic point of view.

Alkaline phosphatase (ALP) is another important indicator of mesenchymal stem cells’ differentiation potential regarding materials designed to interact in a friendly manner with osseous hard tissues like teeth. It indicates cell bioactivity regarding the composite material surface, which is able to promote bio-integration into the host tissue. 

Cell proliferation on the C1 composite surface reveals an average alkaline phosphatase control level of 121.7 u/L, which increases after 1 and 3 days of proliferation to 209.2 u/L. The statistical analysis in Figure 5b reveals an important significance of these values indicating the incipient stage before cell differentiation. The low value of ALP at this early stage is in good agreement with stem cell pluripotency, which agrees with the literature [50,51]. Optical and SEM microstructural investigations reveal physical cell differentiation in the presence of composite samples after 5 and 7 days of proliferation. This fact correlates with the significant increase in ALP level to 279.4 u/L. It indicates an osteoinductive behavior of the differentiated cells that was induced by the C1 sample. Statistical analysis evidence significant difference between the two stages observed.

On the other side, Figure 5c evidences an average alkaline phosphatase control level of 182 u/L that slightly increases after 1 and 3 days of proliferation to a value of about 193 u/L for the C2 sample. Statistical analysis does not reveal any relevant differences in this group indicating an initial stage of MSCs’ pluripotency. The ALP level significantly increases after 5 and 7 days of co-culture, respectively, having the same statistical relevance. It indicates that the C2 sample promotes cell differentiation observed via SEM and optical microscopy as well as corresponding osteoinductive behavior. Statistical observation reveals a clear separation between the observed stages of cell evolution.

## 3. Discussion

SEM microscopy reveals that the filler amount has an important influence on the composite’s microstructure (Figure 1a,b). A higher mineral filler amount within the C1 sample ensures better disintegration of the polycaprolactone core shell and a good distribution of the particles within the polymeric matrix. The importance of filler distribution is also reported in the literature [52,53]. Targeted filler delivery assures optimal lamination of the BaO filler particles and proper embedding of the hydroxyapatite and silanized quartz nanoparticles. EDX spectra evidenced filler elements such as Si, Ba, P and Ca on the sample surface, proving that they were properly released onto the polymeric matrix, ensuring the optimal desired distribution. Some superficial cracks are observed for C1 sample, but they do not penetrate the composite bulk in depth.

Less mineral filler within the C2 sample reduces polycaprolactone envelope braking during composite polymerization, assuring a more progressive embedding of the mineral filler particles into a very well-mixed structure without local segregations and superficial cracks. The fact is sustained by the EDX spectrum that evidences the characteristic elements of the filler: mainly Si and Ba due to nano silica and barium glass, but also P and Ca due to the nHA contents. SEM images in Figure 1c,d also reveal the presence of significant fragments belonging to the polycaprolactone envelope which are well embedded into the polymer matrix, assuring the good cohesion of the composite.

FTIR spectra within Figure 2 evidenced effective incorporation of the mineral filler in both C1 and C2 samples and reveals Bis-GMA stabilization due to the presence of UDMA monomers, which stabilize the carboxylic bonds within the polymeric matrix, controlling viscosity during the polymerization process to assure an optimal and uniform distribution of filler particles in the composite bulk. This fact sustained by the homogeneous aspect of microstructural details. 

Hydroxyapatite is one of the most important constituents of human hard tissues. Nanostructured crystals are embedded with collagen and other protein materials forming different bone tissues [54,55]. It is also reported as a bone implant surface bio-functionalization agent enhancing the bio-response and implant integration in the living body [56,57]. Thus, the uniform distribution of nanostructured hydroxyapatite clusters within C1 and C2 composites leads to a significant cell proliferation after 1 and 3 days of culture as revealed through SEM and optical microscopy. The general aspect of the cells is spindle-like, and central body size is of moderate width around 15–30 µm and a length of about 20–40 µm. Cell density decreases after 5 days of culture due to their natural growth process. Microscopy evidences significantly increased sizes: width of about 27 μm and length of 45 μm, as well as morphological differentiations such local flattening and shape modifications; e.g., elongated, starred and some rounded shapes are observed. Cell differentiation is induced by the co-culture medium’s exposure to the composite samples that stimulated their osteoinductive behavior, a fact sustained by the significantly increased level of P and Ca in the elemental composition measured after 7 days of co-culture. Both composites present good cell proliferation, but C2 proves to be more effective than C1 due to the better distribution of nano-hydroxyapatite clusters within its structure.

The proliferation of mesenchymal stem cells of palatal origin on the composite surface reveals excellent cell viability, proving low cytotoxicity. Cell viability presents a slow decrease along with proliferation time, but the measured values are still excellent, as observed in Figure 5a. Literature data indicate a moderate generation of alkaline phosphatase (ALP) due to the pluripotent state of the MSCs associated with their spindle aspect [50,51]. Statistical analysis of the data in Figure 5b,c proves that the incipient stage lasts up to the third day of co-culture. Cell differentiation is observed from 5 days of co-culture, implying significant generation of ALP. It represents the second stage of ALP variation, and their increase was regarded as a distinct stage via the statistical analysis of the variation in Figure 5b,c. The increase in P and Ca amounts after 7 days of co-culture correlated with an increase in ALP level, indicating that the composite samples stimulate the osteoinductive behavior of MSCs trough the differentiation process. This fact indicates that these experimental materials are compatible with the living body. Data in the literature report an increased level of alkaline phosphatase when the implant bio-integrates; the ALP peak usually occurs at 14 days after in vivo implantation [58,59]. It might facilitate the high-efficacy integration of composite cement into the teeth structure, although this requires further in vivo investigation for our composite samples.

The correlation of experimental results shows that cell proliferation density Is favored by the uniform distribution of the nano-hydroxyapatite clusters within the composite, which induces the differentiation process within MSCs from 5 to 7 days of co-culture and respectively stimulates their osteoinductive behavior. Thus, C2 proves to be more effective than C1, but the relatively high amount of bioactive minerals within C1 samples conducts a significantly greater cell viability and alkaline phosphatase response. Therefore, mineral filler distribution and amount play a key role in the composites’ restorative success, and in consequence, the null hypothesis is completely rejected.

## 4. Materials and Methods

### 4.1. Composite Cement Sample Preparation

The experimental composite cements were prepared in the laboratories of Raluca Ripan Institute for Research in Chemistry (ICCRR, Cluj-Napoca, Romania).

The polymeric matrix contains Bis-GMA (2,2-bis[p-(2’-hydroxy-3’-metacryloxy propoxy)phenyl]-propane), UDMA (urethane dimethacrylate), HEMA (Hydroxyethyl methacrylate) and PEG 400 (polyethylene glycol); all substances were analytical grade and purchased from Sigma Aldrich, Darmstadt, Germany.

The mineral filler contains BaO glass, silanized quartz nanoparticles and hydroxyapatite nanoparticles purchased from Sigma Aldrich, Darmstadt, Germany. They were encapsulated into polycaprolactone (analytical grade Sigma Aldrich, Darmstadt, Germany) microcapsules produced in our laboratory using buffalo whey (Advanced Materials, Inc., Westerlo Oevel, Belgium) as a nanoparticle dispersion environment.

We prepared two composite cements using the same polymer matrix and filler. The difference consists in the matrix/filler ratio: C1 has a ratio of 67/33% and C2 has a ratio of 69/31%. The composition was mixed using a mechanical homogenizer for 10 min. The photo-polymerization process was initiated using a camphorquinone photoinitiator (CQ) and 2-(Dimethylamino)ethyl methacrylate (DMAEM) (Sigma Aldrich, Darmstadt, Germany). A photo-polymerization lamp was used (Elipar Deep Cure-L 3 M produced by 3M Oral Care Company, Saint Paul, MN, USA). The photo-polymerization lamp was placed as close as possible to the disks’ surface. Both composites were molded into disk-shaped samples with a diameter of 5 mm and a thickness of 0.5 mm.

### 4.2. Palatal Mesenchymal Stem Cell Co-Cultivation and MTT Assay

The palatal mesenchymal stem cells used in the current research were obtained from the Biotechnology Laboratory of the University of Agricultural Sciences and Veterinary Medicine (Cluj-Napoca, Romania) and has the approval of Bioethical Commission No. 352 from 12 December 2022. The stem cell line was stabilized by our team and was previously published [60].

Palatal-tissue-derived mesenchymal cells were cultured in a simple propagation medium of DMEM/F12 (Sigma-Aldrich, Darmstadt, Germany) supplemented with 10% FCS (fetal bovine serum, Sigma-Aldrich), 1% antibiotic-antimycotic (Gibco), 1% glutamax 1× (Sigma-Aldrich, Darmstadt, Germany) and 1% NEA (non-essential amino acids, Gibco). To evaluate the biomaterials, 12-well plates (Thermo Fischer Scientific, Waltham, MA, USA) were used. The concentration of the palatal cells used was 1 × 10^5^. The bioactive potential of bioglass-based composites (C1 and C2) was evaluated through co-culture techniques using inserts in which the composite samples were properly placed, and afterwards, the cells were added on their surface in simple propagation medium. After the desired proliferation time (3, 5 and 7 days of co-culture), the MTT (4,5-dimethylthiazol-2-yl)-2,5-diphenyltetrazolium) assay was used to assess cytotoxicity and degree of proliferation according to the ISO 10993-5:2009 protocols. The MTT (4,5-dimethylthiazol-2-yl)-2,5-diphenyltetrazolium) assay (Sigma-Aldrich, Darmstadt, Germany) measures cellular metabolic activity as an indicator of cell viability, proliferation and cytotoxicity. This colorimetric assay is based on the reduction of tetrazolium salts to purple formazan crystals by cells that are metabolically active. Viable cells contain NAD(P)H-dependent oxidoreductase enzymes that reduce MTT to formazan. Insoluble formazan crystals were dissolved with dimethylsulfoxide (DMSO), and the absorbance of the chromogenic reaction was evaluated using a spectrophotometer at a wavelength of 450 nm.

Alkaline phosphatase (ALP) level was measured on request in the clinical laboratory of the Veterinary Medicine Faculty in Cluj-Napoca, Romania.

Both MTT assay and ALP data were statistically processed using ANOVA one-way followed by Tukey post-hoc tests using Origin 2019b software (Microcal Corporation, Northampton, MA, USA). The significance level was set at 0.05; thus, *p* < 0.05 indicates that significant statistical differences occur and *p* > 0.05 means that there are no significant differences between tested group elements.

### 4.3. Microscopy Techniques

Optical microscopy investigation was effectuated using a transmitted light biological microscope (ZEISS Axioscope 5, Oberocken, Germany).

Scanning Electron Microscopy was effectuated using a Hitachi SU8230 Scanning Electron Microscope, Tokyo, Japan, at an acceleration voltage of 30 kV in low-vacuum mode without sample metallization. Elemental analysis was effectuated with an Energy-Dispersive Spectroscopy (EDS) detector: X-Max 1160 EDX (Oxford Instruments, Oxford, UK). The culture medium within wells was removed before SEM investigation and afterwards rinsed with ultrapure water and stored under a desiccator for 1 h until their surface was completely dry. 

Fourier-transform infrared spectroscopy (FTIR) was performed using an FTIR 610 spectrometer (Jasco Corporation, Tokyo, Japan) in the wavenumber range of 4000–400 cm^−1^, using the potassium bromide pellet technique. Each spectrum was registered at a resolution of 4 cm^−1^ and represents the average of 100 scans.

## 5. Conclusions

Controlled filler release through photo-polymerization was properly achieved using polycaprolactone microcapsules. The uniformity of nanostructured filler particles is slightly increased for C2 samples, which is more favorable for palatal mesenchymal stem cell cluster proliferation. Cell viability strongly depends on the amount of bioactive filler. The best results of the MTT assay show that C1 has a greater value of MSC viability. The alkaline phosphatase (ALP) level also was increased for the higher amount of bioactive filler. Two stages occur: the initial stage up to 3 days of co-culture is characterized by an average generation of ALP associated with the pluripotency of the MSCs, and the second stage (from 5 to 7 days of co-culture) is characterized by a significant increase in the ALP level due to MSC differentiation induced by the composite samples that stimulate their osteoinductive behavior. It indicates that both C1 and C2 samples are viable candidates for further in vivo tests.

## Figures and Tables

**Table 1:** Elemental composition obtained via SEM images for composite samples.

Samples	Identified Elements, Wt.%.					
	C	O	Si	P	Ca	Ba
C1	34.05	16.96	24.13	3.07	2.98	18.81
C2	33.98	21.48	23.91	2.32	2.26	16.05

**Table 2:** Elemental composition obtained via SEM images after 7 days of co-culture.

Samples	Identified Elements, Wt.%.					
	C	O	Si	P	Ca	Ba
C1—7 days	31.01	12.64	25.46	6.11	5.04	19.74
C2—7 days	27.54	18.32	26.63	5.16	5.22	17.13

## Data Availability

The research data are available on request from the corresponding author.

## References

[1] Jurak M., Wiącek A.E., Ładniak A., Przykaza K., Szafran K. (2021). What affects the biocompatibility of polymers?. Adv. Colloid Interface Sci..

[2] Ratner B.D., Schoen F.J., Lemons J.E. (1996). Biomaterials Science: An Introduction to Materials in Medicine.

[3] Vert M., Doi Y., Hellwich K.H., Hess M., Hodge P., Kubisa P., Rinaudo M., Schue F. (2012). Terminology for biorelated polymers and applications (IUPAC Recommendations 2012). Pure Appl. Chem..

[4] Azevedo H.S., Mata A. (2022). Embracing complexity in biomaterials design. Biomater. Biosyst..

[5] Simionescu B.C., Ivanov D., Antoniac I.V. (2015). Natural and synthetic polymers for designing composite materials. Handbook of Bioceramics and Biocomposites.

[6] Bonferoni M.C., Caramella C., Catenacci L., Conti B., Dorati R., Ferrari F., Genta I., Modena T., Perteghella S., Rossi S. (2021). Biomaterials for Soft Tissue Repair and Regeneration: A Focus on Italian Research in the Field. Pharmaceutics.

[7] Conte R., Di Salle A., Riccitiello F., Petillo O., Peluso G., Calarco A. (2018). Biodegradable polymers in dental tissue engineering and regeneration. AIMS Mater. Sci..

[8] Calori I.R., Braga G., de Jesus P.D.C.C., Bi H., Tedesco A.C. (2020). Polymer scaffolds as drug delivery systems. Eur. Polym. J..

[9] Anju S., Prajitha N., Sukanya V.S., Mohanan P.V. (2020). Complicity of degradable polymers inhealth-care applications. Mater. Today Chem..

[10] Collignon A.M., Lesieur J., Vacher C., Chaussain C., Rochefort G.Y. (2017). Strategies Developed to Induce, Direct, and Potentiate Bone Healing. Front. Physiol..

[11] Chisnoiu R., Moldovan M., Pastrav O., Delean A., Chisnoiu A.M. (2016). The influence of three endodontic sealers on bone healing–An experimental study. Folia. Morphol..

[12] Lee S.-Y., Wu S.-C., Chen H., Tsai L.-L., Tzeng J.-J., Lin C.-H., Lin Y.-M. (2018). Synthesis and Characterization of Polycaprolactone-Based Polyurethanes for the Fabrication of Elastic Guided Bone Regeneration Membrane. BioMed Res. Int..

[13] Luo S., Zhu W., Liu F., He J. (2016). Preparation of a Bis-GMA-Free Dental Resin System with Synthesized Fluorinated Dimethacrylate Monomers. Int. J. Mol. Sci..

[14] Ahovuo-Saloranta A., Hiiri A., Nordblad A., Mäkelä M., Worthington H.V. (2017). Pit and fissure sealants for preventing dental decay in the permanent teeth of children and adolescents. Cochrane Database Syst. Rev..

[15] Haugen H.J., Marovic D., Par M., Le Thieu M.K., Reseland J.E., Johnsen G.F. (2020). Bulk Fill Composites Have Similar Performance to Conventional Dental Composites. Int. J. Mol. Sci..

[16] Barszczewska-Rybarek I.M., Chrószcz M.W., Chladek G. (2020). Novel Urethane-Dimethacrylate Monomers and Compositions for Use as Matrices in Dental Restorative Materials. Int. J. Mol. Sci..

[17] Raszewski Z., Brząkalski D., Derpeński Ł., Jałbrzykowski M., Przekop R.E. (2022). Aspects and Principles of Material Connections in Restorative Dentistry—A Comprehensive Review. Materials.

[18] Limberger K.M., Westphalen G.H., Menezes L.M., Medina-Silva R. (2011). Cytotoxicity of orthodontic materials assessed by survival tests in Saccharomyces cerevisiae. Dent. Mater..

[19] Yoshinaga K., Yoshihara K., Yoshida Y. (2021). Development of new diacrylate monomers as substitutes for Bis-GMA and UDMA. Dent. Mater..

[20] Szczesio-Wlodarczyk A., Domarecka M., Kopacz K., Sokolowski J., Bociong K. (2021). An Evaluation of the Properties of Urethane Dimethacrylate-Based Dental Resins. Materials.

[21] Moszner N., Fischer U.K., Angermann J., Rheinberger V. (2008). A partially aromatic urethane dimethacrylate as a new substitute for Bis-GMA in restorative composites. Dent. Mater..

[22] Ma T.Y., Hollander D., Krugliak P., Katz K. (1990). PEG 400, a hydrophilic molecular probe for measuring intestinal permeability. Gastroenterology.

[23] Mohl S., Winter G. (2004). Continuous release of rh-interferon alpha-2a from triglyceride matrices. J. Control. Release.

[24] Hench L.L. (2015). The future of bioactive ceramics. J. Mater. Sci. Mater. Med..

[25] Crush J., Hussain A., Seah K.T.M., Khan W.S. (2021). Bioactive Glass: Methods for Assessing Angiogenesis and Osteogenesis. Front. Cell Dev. Biol..

[26] Gloria A., Russo T., Rodrigues D.F.L., D’Amora U., Colella F., Improta G., Triassi M., De Santis R., Ambrosio L. (2016). From 3D hierarchical scaffolds for tissue engineering to advanced hydrogel-based and complex devices for in situ cell or drug release. Procedia CIRP.

[27] Rasouli R., Barhoum A., Uludag H. (2018). A review of nanostructured surfaces andmaterialsfor dental implants: Surface coating, patterning and functionalization for improved performance. Biomater. Sci..

[28] Kumar S., Nehra M., Kedia D., Dilbaghi N., Tankeshwar K., Kim K.-H. (2020). Nanotechnologybased biomaterials for orthopaedic applications: Recent advances and future prospects. Mater. Sci. Eng. C Mater. Biol. Appl..

[29] Liu X., Chu P.K., Ding C. (2010). Surface nano-functionalization of biomaterials. Mater. Sci. Eng. R.

[30] Yang P., Ren J., Yang L. (2023). Nanoparticles in the New Era of Cardiovascular Therapeutics: Challenges and Opportunities. Int. J. Mol. Sci..

[31] Undin J., Finne-Wistrand A., Albertsson A.-C. (2014). Adjustable degradation properties andbiocompatibility of amorphous and functional poly(ester-acrylate)-based materials. Biomacromolecules.

[32] Gu Q., Cuevas E., Ali S.F., Paule M.G., Krauthamer V., Jones Y., Zhang Y. (2019). An Alternative In Vitro Method for Examining Nanoparticle-Induced Cytotoxicity. Int. J. Toxicol..

[33] Sanità G., Carrese B., Lamberti A. (2020). Nanoparticle surface functionalization: How to improvebiocompatibility and cellular internalization. Front. Mol. Biosci..

[34] Mousavinasab S.M. (2011). Biocompatibility of composite resins. Dent. Res. J..

[35] Pătroi D., Gociu M., Prejmerean C., Colceriu L., Dumitrescu L.S., Moldovan M., Naicu V. (2013). Assessing the biocompatibility of a dental composite product. Rom. J. Morphol. Embryol..

[36] Wataha J.C. (2001). Principles of biocompatibility for dental practitioners. J. Prosthet. Dent..

[37] Kawai M.Y., Ozasa R., Ishimoto T., Nakano T., Yamamoto H., Kashiwagi M., Yamanaka S., Nakao K., Maruyama H., Bessho K. (2022). Periodontal Tissue as a Biomaterial for Hard-Tissue Regeneration following *bmp-2* Gene Transfer. Materials.

[38] Xu X.Y., Li X., Wang J., He X.T., Sun H.H., Chen F.M. (2019). Concise review: Periodontal tissue regeneration using stem cells: Strategies and translational considerations. Stem Cells Transl. Med..

[39] Han J., Menicanin D., Gronthos S., Bartold P.M. (2014). Stem cells, tissue engineering and periodontal regeneration. Aust. Dent. J..

[40] Lawrence L.M., Salary R., Miller V., Valluri A., Denning K.L., Case-Perry S., Abdelgaber K., Smith S., Claudio P.P., Day J.B. (2023). Osteoregenerative Potential of 3D-Printed Poly *ε*-Caprolactone Tissue Scaffolds In Vitro Using Minimally Manipulative Expansion of Primary Human Bone Marrow Stem Cells. Int. J. Mol. Sci..

[41] Zhao Y., Zhang H., Hong L., Zou X., Song J., Han R., Chen J., Yu Y., Liu X., Zhao H. (2023). A Multifunctional Dental Resin Composite with Sr-N-Doped TiO_2_ and n-HA Fillers for Antibacterial and Mineralization Effects. Int. J. Mol. Sci..

[42] Ansari M.A., Jahan N. (2021). Structural and Optical Properties of BaO Nanoparticles Synthesized by Facile Co-precipitation Method. Mater. Highlights.

[43] Osipov A.A., Osipova L.M., Hruška B., Osipov A.A., Liška M. (2019). FTIR and Raman spectroscopy studies of ZnO-doped BaO 2B_2_O_3_ glass matrix. Vib. Spectrosc..

[44] Gheisari H., Karamian E., Abdellahi M. (2015). A novel hydroxyapatite–Hardystonite nanocomposite ceramic. Ceram. Int..

[45] Koupadi K., Boyatzis S.C., Roumpou M., Kalogeropoulos N., Kotzamani D. (2021). Organic remains in early Christian Egyptian metal vessels: Investigation with fourier transform infrared spectroscopy and gas chromatography–mass spectrometry. Heritage.

[46] Farcas I.A., Dippong T., Petean I., Moldovan M., Filip M.R., Ciotlaus I., Tudoran L.B., Borodi G., Paltinean G.A., Pripon E. (2023). Material Evidence of Sediments Recovered from Ancient Amphorae Found at the Potaissa Roman Fortress. Materials.

[47] Rusca M., Rusu T., Avram S.E., Prodan D., Paltinean G.A., Filip M.R., Ciotlaus I., Pascuta P., Rusu T.A., Petean I. (2023). Physicochemical Assessment of the Road Vehicle Traffic Pollution Impact on the Urban Environment. Atmosphere.

[48] Moldovan M., Dudea D., Cuc S., Sarosi C., Prodan D., Petean I., Furtos G., Ionescu A., Ilie N. (2023). Chemical and Structural Assessment of New Dental Composites with Graphene Exposed to Staining Agents. J. Funct. Biomater..

[49] Mazilu A., Popescu V., Sarosi C., Dumitrescu R.S., Chisnoiu A.M., Moldovan M., Dumitrescu L.S., Prodan D., Carpa R., Gheorghe G.F. (2021). Preparation and In Vitro Characterization of Gels Based on Bromelain, Whey and Quince Extract. Gels.

[50] Stefkova K., Prochazkova J., Pachernik J. (2015). Alkaline Phosphatase in Stem Cells. Stem Cells Int..

[51] Domínguez L.M., Bueloni B., Cantero M.J., Albornoz M., Pacienza N., Biani C., Luzzani C., Miriuka S., García M., Atorrasagasti C. (2023). Chromatographic Scalable Method to Isolate Engineered Extracellular Vesicles Derived from Mesenchymal Stem Cells for the Treatment of Liver Fibrosis in Mice. Int. J. Mol. Sci..

[52] Tiskaya M., Shahid S., Gillam D., Hill R.G. (2021). The use of bioactive glass (BAG) in dental composites: Critical review. Dent. Mater..

[53] Bin-Jardan L.I., Almadani D.I., Almutairi L.S., Almoabid H.A., Alessa M.A., Almulhim K.S., AlSheikh R.N., Al-Dulaijan Y.A., Ibrahim M.S., Al-Zain A.O. (2023). Inorganic Compounds as Remineralizing Fillers in Dental Restorative Materials: Narrative Review. Int. J. Mol. Sci..

[54] Rashid U., Becker S.K., Sponder G., Trappe S., Sandhu M.A., Aschenbach J.R. (2023). Low Magnesium Concentration Enforces Bone Calcium Deposition Irrespective of 1,25-Dihydroxyvitamin D_3_ Concentration. Int. J. Mol. Sci..

[55] Lv S., Yuan X., Xiao J., Jiang X. (2023). Hemostasis-osteogenesis integrated Janus carboxymethyl chitin/hydroxyapatite porous membrane for bone defect repair. Carbohydr. Polym..

[56] Kazimierczak P., Wessely-Szponder J., Palka K., Barylyak A., Zinchenko V., Przekora A. (2023). Hydroxyapatite or Fluorapatite—Which Bioceramic Is Better as a Base for the Production of Bone Scaffold?—A Comprehensive Comparative Study. Int. J. Mol. Sci..

[57] Makishi S., Watanabe T., Saito K., Ohshima H. (2023). Effect of Hydroxyapatite/β-Tricalcium Phosphate on Osseointegration after Implantation into Mouse Maxilla. Int. J. Mol. Sci..

[58] Chai Q., Xu H., Xu X., Li Z., Bao W., Man Z., Li W. (2023). Mussel-inspired alkaline phosphatase-specific coating on orthopedic implants for spatiotemporal modulating local osteoimmune microenvironment to facilitate osseointegration. Colloids Surf. B Biointerfaces.

[59] Zhuravleva I.Y., Karpova E.V., Dokuchaeva A.A., Titov A.T., Timchenko T.P., Vasilieva M.B. (2023). Calcification of Various Bioprosthetic Materials in Rats: Is It Really Different?. Int. J. Mol. Sci..

[60] Páll E., Florea A., Soriţău O., Cenariu M., Petruţiu A.S., Roman A. (2015). Comparative Assessment of Oral Mesenchymal Stem Cells Isolated from Healthy and Diseased Tissues. Microsc. Microanal..

